# Applicability of a Dexterity-Enhancing Handheld Robot for 360° Endoscopic Skull Base Approaches: An Exploratory Cadaver Study

**DOI:** 10.1227/ons.0000000000001582

**Published:** 2025-04-28

**Authors:** Joachim Starup-Hansen, Dan Zimelewicz Oberman, John G. Hanrahan, Emmanouil Dimitrakakis, Hani J. Marcus, Joao Paulo Almeida

**Affiliations:** ‡ Victor Horsley Department of Neurosurgery, National Hospital for Neurology and Neurosurgery, London, UK;; § Wellcome/EPSRC Centre for Interventional and Surgical Sciences, University College London, London, UK;; ‖ Department of Neurological Surgery, Mayo Clinic, Jacksonville, Florida, USA;; ¶ Panda Surgical Ltd, London, UK;; # Department of Neurosurgery, Indiana University, Indianapolis, Indiana, USA

**Keywords:** Cadaver, Endoscopy, Skull base, Robotics

## Abstract

**BACKGROUND AND OBJECTIVES:**

Endoscopic skull base surgery aims to reduce surgical morbidity by minimizing tissue manipulation and exposure. However, the anatomic constraints posed by the narrow surgical corridors and constrained operative workspace present technical challenges due to reduced dexterity. This study evaluates the applicability of a novel dexterity-enhancing handheld robot for endoscopic skull base approaches.

**METHODS:**

The robotic system is comprised of interchangeable articulated end-effectors coupled to a handheld controller. Two attending skull base neurosurgeons and 2 neurosurgery residents performed 8 skull base approaches on cadaveric specimens, spanning anterior, anterolateral, lateral, posterolateral, and posterior approaches. Conventional instruments were used to expose anatomic landmarks, followed by intraoperative tasks using the handheld robot. Participants were interviewed during the procedures to assess the robot's feasibility (ability to safely reach and perform its objective of manipulating tissue at the operative site) and usefulness (ability to perform desired objectives well).

**RESULTS:**

The handheld robotic system was tested across 8 endoscopic skull base approaches, achieving feasibility in all cases. Superior workspace reach compared with standard instruments was demonstrated in 6 of 8 approaches. Tissue manipulation was satisfactory in all approaches. All surgeons reported that the current or a future device prototype could be useful across all 8 approaches. The most frequently cited advantage was the expanded dextrous workspace reach provided by the articulated end-effectors, particularly in approaches with long working channels, such as the endonasal approach. However, the robot encountered difficulties in transcranial approaches (trans-sylvian and subtemporal) due to the lack of shorter, curved shafts, which impaired visualization.

**CONCLUSION:**

The handheld robotic system demonstrated applicability across various endoscopic skull base approaches, offering increased dextrous workspace and effective tissue manipulation capabilities. Overall, this study supports the potential of handheld robots in endoscopic skull base surgery while highlighting the need for iterative development to optimize instrument design and functionality.

Minimally invasive approaches to the skull base are increasingly common, aiming to reduce surgical morbidity through reduced exposure and tissue manipulation.^1^ The advent of endoscopic technology offers potentially less invasive approaches to these complex anatomic regions, enabling surgeons to navigate the narrow corridors of the skull base with improved wide-angle visualization.^2-4^ However, although endoscopic approaches to the skull base are less invasive, they also present considerable technical challenges. First, the currently available instruments lack articulation, resulting in surgeons needing to manipulate tissue in a coaxial direction to the viewing angle, limiting their dextrous workspace reach.^4-7^ Second, the approaches consist of narrow operative corridors, which impose fulcrum effects on surgical instruments, challenging surgeons in making precise movements.^4-7^ Ultimately, these instrument limitations restrict what is technically feasible through endoscopic approaches, such as in the endonasal approach to the para-sellar region, where surgeons are limited in their reach of tumors which extend significantly beyond the sella turcica. Therefore, to optimize the benefits of minimally invasive skull base approaches, there is a pressing need for the development of innovative surgical instruments to meet these challenges.

In previous work, we introduced and evaluated a novel, dexterity-enhancing handheld robotic instrument featuring articulated instruments designed to overcome the inherent challenges associated with endoscopic neurosurgery.^8,9^ Most recently, the robotic prototype underwent a preclinical cadaveric assessment of its feasibility for an endoscopic endonasal approach to the sellar and parasellar regions, demonstrating its potential to overcome many of the inherent difficulties of skull base surgery.

Building on this foundation, this study aims to explore the broader applicability of this robotic technology, assessing its feasibility and potential usefulness for comprehensive 360° endoscopic endonasal and transcranial approaches to the skull base. Beyond this, it aims to identify relevant design updates that may augment the device's applicability to these approaches. This article details our findings in a cadaveric study and discusses the implications of integrating such robotic assistance into the wider skull base approaches.

## METHODS

### Study Design

Two skull base neurosurgeons and 2 intermediate-level neurosurgical residents with prior skull base training were recruited from a single, independent neurosurgical unit, with no ties to the development or manufacturer of the robotic device. In accordance with local policy, formal institutional review board approval was not required for cadaveric studies. Appropriate consent was obtained for the publication of the cadaveric images.

Participants performed various skull base approaches on 6 cadaveric specimens, accessing the anterior, middle, and posterior cranial fossae. Approaches included anterior approaches (endonasal,^4^ transorbital^10^) and antero-lateral approaches (supraorbital,^11^ pterional-transylvian^12^), lateral (subtemporal^13^) and posterolateral (retrosigmoid,^13^ far lateral) approaches,^14^ and posterior approaches (suboccipital^15^). They used conventional instruments to achieve satisfactory anatomic exposure and then assessed the feasibility and usefulness of a handheld robot for completing intraoperative tasks. Feasibility was based on the robot's ability to safely navigate and manipulate tissue, while usefulness was evaluated through workspace reach, tissue manipulation, and future clinical potential. Interviews were conducted to evaluate these factors (Table 1).

**TABLE 1.** Procedural Questionnaire (Evaluated for Each Surgical Approach after Exposure)

Domain	Question
Feasibility	Does the robot safely fit within the anatomic confines of the surgical corridor?
Usefulness	Workspace-reach: After a satisfactory approach and exposure, please use the robot to reach relevant anatomic landmarks. Did you find the robot useful in reaching tissue?
	Tissue manipulation: Once you have achieved satisfactory exposure and are able to reach anatomic areas of interest, please manipulate soft tissue close to the surgical area of interest. Did you find the robot useful in manipulating soft tissue?
	Overall, could you envisage a future prototype of the handheld robot being a useful adjunct for the given approach? If so, for what intraoperative task(s)/actions?
Improvements	Is the shaft length appropriate for the current approach? (ie, not too long or too short?)
	Which other end effectors could you envisage being useful for this approach?
	Are there any other modifications to the robot that would make it more suitable for the current approach?

### Instruments

**FIGURE 1.**
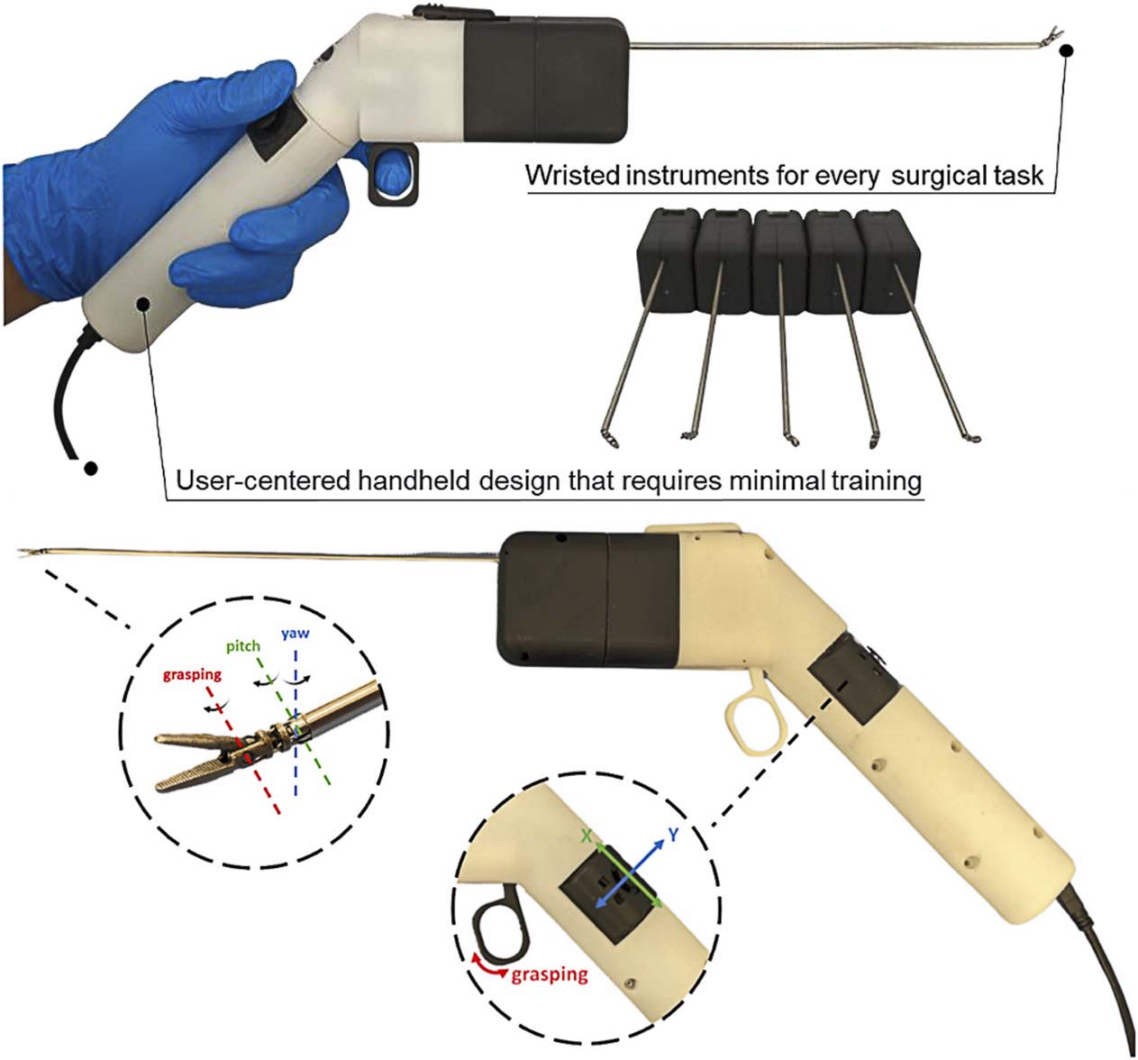
Surgical handheld system: The white component is the handheld controller, whereas the black component with the silver shaft that is connected to the controller is one of the interchangeable instruments that can be replaced with any of the other instruments below it. Controller is connected to the console through a cable. Pitch, yaw, and grasping axes around which the flexible instrument tip rotates and the corresponding motions of the joystick and trigger.

**FIGURE 2.**
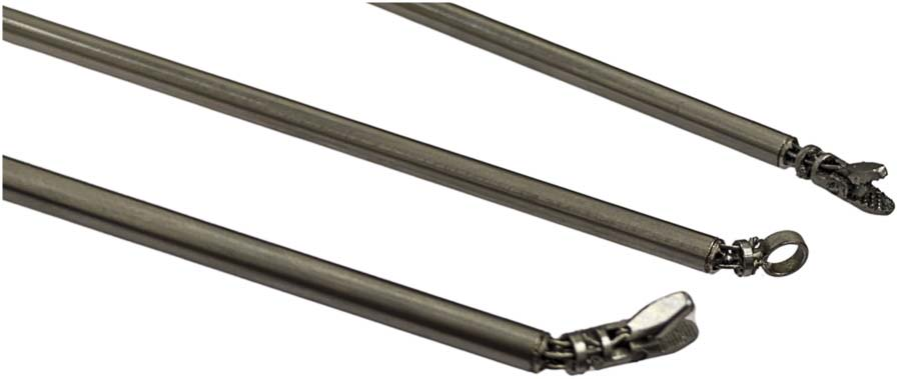
Depicting the available articulated end-effectors. From top to bottom: needle holder, ring-curette, and grasper.

The robotic system comprises a handheld device with a joystick and trigger (Figure 1) controlling a 3-mm diameter interchangeable wristed instruments such as graspers and curettes (Figure 2). offers a demonstration of the device's functionality using the grasper end-effector. The joystick moves the end-effector in pitch and yaw (Figure 1), whereas the trigger controls the grasper's opening and closing. The product is investigational and therefore not labelled for used under discussion. Visualization was provided by 0° and 30° endoscopes, alongside manual neurosurgical instruments.

## RESULTS

**FIGURE 3.**
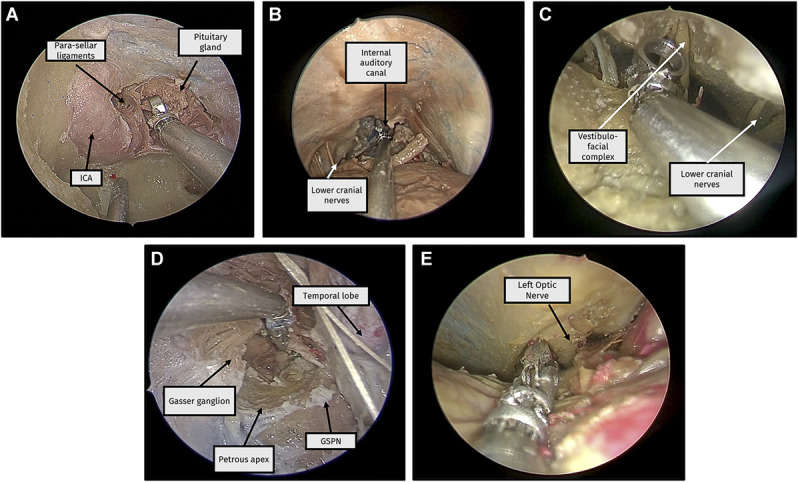
Selection of endoscopic images of the handheld robot manipulating tissue at the operative site. **A**, Endonasal approach, **B**, retrosigmoid approach, **C**, far lateral approach, **D**, transorbital approach, and **E**, supraorbital approach. GSPN, greater superficial petrosal nerve; ICA, internal carotid artery.

The robot was evaluated across 8 skull base approaches. Feasibility was achieved in all 8 approaches, and all surgeons reported that a current or future device prototype could be useful for all 8 approaches (Figure 3). The robot had a useful workspace reach in all 8 approaches, with 6 of 8 being superior to conventional instruments. The robot also demonstrated useful tissue manipulation capabilities in all 8 approaches. Table 2 presents an overview of device limitations.

**TABLE 2.** Robotic Device Limitations

Component	Limitations
End-effectors	• Limited grasping force • Tool tip too large • Tool tip too sharp • Lack of certain end-effectors including: dissectors, feather blades, microscissors, bipolar forceps, Blakesley forceps, dural-knives, vertical curettes, thru-cuts, and suction • Instrument tip misconfigures under angulated strain
Shaft	• Shaft is too long for some approaches • Lack of angled shafts impedes tip visualization in certain angles
Controller	• Trigger control nonintuitive • Nonintuitive end-effector control if device rotated around z-axis • Device too heavy • Pistol shaped grip too bulky

### Anterior Approaches

#### Expanded Endonasal Approach

In the extended endonasal approach, after performing the sphenoidotomy and drilling the sellar and parasellar regions, the anatomic structures of the sella, including the pituitary gland, the segments of the internal carotid artery, the limbus, and the planum sphenoidale, were exposed. The cadaver was not sufficiently pneumatized to reach the lateral recess of the sphenoid sinus. After the opening of the sellar dura, the robot was used to conduct intradural dissection, separating the pituitary from the medial wall of the cavernous sinus while preserving the capsular plane of the gland. Subsequently, after opening the anterior wall of the cavernous sinus, the robot was used to dissect the para-sellar and carotid-clinoid ligaments, aiming to remove the medial wall of the cavernous sinus.

In the operator interviews, users deemed the device feasible for the extended endonasal approach and could envisage the robot being useful. The robot exhibited workspace reach superior to conventional instruments and useful tissue manipulation capabilities. Highlighted limitations of the robot for the transsphenoidal approach related to the end-effector's insufficient grasping forces, restricted diagonal reach, joint misconfiguration during angulated strains, and the lack of certain end-effectors (dissectors, Blakesley forceps, knife, scissors, vertical curettes, and thru-cuts). In addition, it was noted that the straight shaft of the device hindered deep access to the intracranial fossae.

#### Endoscopic Transorbital Approach

In the endoscopic transorbital approach, the robot assisted the peeling of the middle fossa from anterior to posterior, exposing the floor of the middle fossa and the petrous apex. In addition, relevant anatomy including the trigeminal nerve, trochlear nerve, oculomotor nerve, middle meningeal artery, greater superficial petrosal nerve, and the internal carotid artery was exposed. After partially drilling the petrous bone, the robot was extremely useful for reaching the posterior fossa region due to the difficult angles involved. The robot's maneuverability allowed for various types of movements simultaneously, eliminating the need to constantly switch instruments.

**FIGURE 4.**
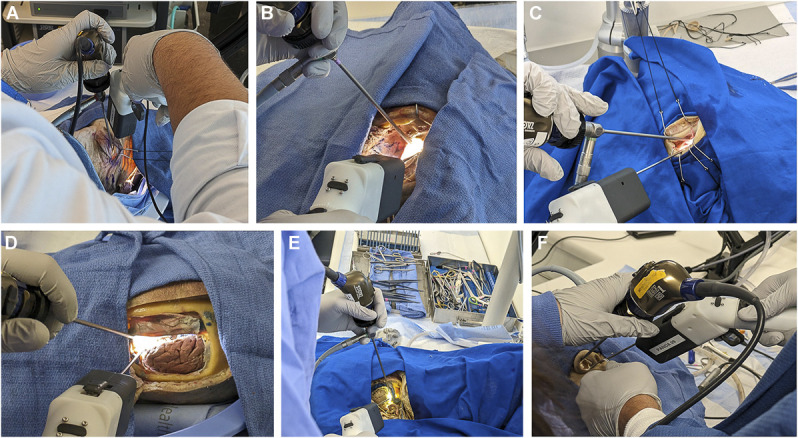
Demonstrating the use of the robot across a variety of endoscopic skull base approach. **A**, Transorbital approach, **B**, supraorbital approach, **C**, retrosigmoid approach, **D**, suboccipital approach, **E**, far lateral approach, and **F**, endonasal approaches.

All users found the device feasible for the approach and could envisage a future prototype of the device as being used for the approach. In addition, the robot demonstrated superior workspace reach to conventional instruments and useful tissue manipulation capabilities. Limitations included insufficient grasping forces hindering manipulation, the lack of angulated shafts (which impaired workspace reach due to anatomic constraints), and poor physical ergonomics due to awkward hand positioning with the pistol grip controller (Figure 4).

### Antero-Lateral (Supraorbital) Approach

In the supraorbital approach, an eyebrow incision and a small craniotomy of 2 × 2 cm was performed. After opening the dura, operators first attempted dissection of the most anterior portion of the skull base (cribriform plate) using conventional instruments, but efforts were limited by angulation constraints. To overcome this, the robot was used to dissect the region between the olfactory nerve and the most anterior part of the falx. The robot's dexterity and precise control facilitated useful dissection in this area, significantly improving access and maneuverability.

Overall, operators found the current prototype feasible for the supraorbital approach and could envisage it being useful in a clinical setting. The robot demonstrated superior workspace reach to conventional instruments and useful tissue manipulation capabilities. Limitations related to the end-effector's grasping forces and larger-than-ideal tooltips which were also too sharp, risking vascular injury in some contexts. In addition, operators found the shaft length to be longer than necessary and lacking angulation.

### Lateral Approaches

For the pterional (trans-sylvian) and subtemporal approaches, the robot assisted in the dissection of the sylvian fissure, expanding the corridors to reach the basal cisterns. In the subtemporal approach, the robot facilitated the dissection and access to the crural and ambient cisterns.

In the surgeon interviews, the current prototype was deemed feasible for the lateral approaches, and it was felt that future editions could be useful. However, workspace reach was inferior to conventional tools, owing to the device's straight shaft, which obstructed visualization of the tip and limited downward angulation of the end-effector. Moreover, end-effectors such as dissectors were felt to be missing from the repertoire.

### Posterolateral

In the retrosigmoid approach, a curvilinear incision was made, followed by a craniotomy that exposed the sigmoid sinus, transverse sinus, and their junction. After opening the dura in a C shape, the robot assisted in opening the cerebellomedullary cistern and subsequently the dissection of the cerebellopontine angle. This facilitated the identification and separation of the lower cranial nerves (accessory nerve, vagus nerve, and glossopharyngeal nerve), as well as the vestibular and facial nerves and the superior segment involving the trigeminal nerve. In addition, the robot was capable of delicate tissue manipulation within the internal auditory canal.

Overall, the robot demonstrated useful workspace reach, superior to conventional instruments, and satisfactory tissue manipulation capabilities. Limitations included the relatively large instrument tips, the lack of certain end-effectors (scissors, dissectors), shafts that were too long and missing angulation, and ergonomic concerns regarding the controller (too heavy and pistol shape too bulky). Overall, it was felt that a future prototype could be useful for the retrosigmoid approach.

In the far-lateral approach, a suboccipital craniotomy was performed and the C1 arch resected. With the assistance of the robot, the lower cranial nerves (including the hypoglossal nerve) and the cervical nerves were exposed, and their relationships with the vertebral artery and the posterior inferior cerebellar artery were delineated. The robot facilitated the dissection of the neurovascular structures at the craniocervical junction, allowing for an angulated approach from inferior to superior, which is one of the primary objectives of this access route. Overall, workspace reach was superior to conventional instruments for the approach, and tissue manipulation was useful. Limitations related to instrument tips being too large and the shaft being too long and lacking angulation.

### Posterior Approaches

In the suboccipital approach, a midline incision with suboccipital craniotomy and subsequent resection of the C1 arch was performed. After opening the dura, access was gained to the cisterna magna and the cerebellomedullary fissures were dissected bilaterally using the robot, identifying the main neurovascular structures. Next, resection was conducted of the tela choroidea and velum medullare, reaching the lateral recess of the fourth ventricle, aiming to expose the entire anatomy of the fourth ventricle. The robot supported the mobilization of the cerebellar tonsils and dissection of the lateral recess of the fourth ventricle (located deep within the field). This was achieved by the precise maneuverability in the confined working space.

In the suboccipital approach, the robot demonstrated a workspace reach superior to that of conventional instruments with useful tissue manipulation capabilities. Device limitations included a longer than necessary shaft, the lack of availability of certain end-effectors (dissectors, scissors, bipolar forceps, and suction), and a bulky handheld controller.

## DISCUSSION

### Principal Findings

**FIGURE 5.**
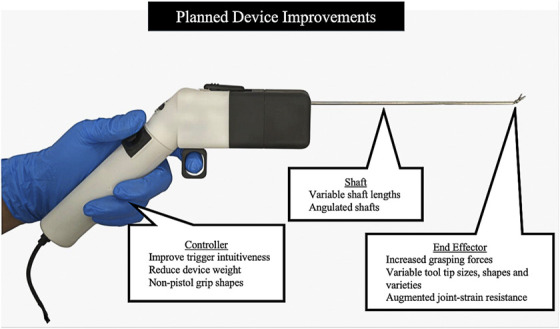
Planned device improvements for the handheld robot.

This study evaluated the feasibility and usefulness of a dexterity-enhancing handheld robot designed for neurosurgery across 8 endonasal endoscopic skull base approaches. The robot was feasible and potentially useful for all approaches studied, allowing for technical modifications. The device improved workspace reach in 6 of 8 approaches and was capable of useful tissue manipulation in all approaches. Surgeons were most positive about the usefulness of the robot in endonasal, postero-lateral (retrosigmoid), and posterior (suboccipital) approaches. While feasible and potentially useful, the robot demonstrated the poorest performance in the trans-sylvian and subtemporal approaches. This demonstrates the value of early-stage device evaluations (guiding device development) yet is ultimately unsurprising, as the robot was custom-built around the constraints of the anterior, endonasal approaches to the sellar and parasellar regions, wherein a long shaft is necessary to facilitate distal tissue manipulation beyond the long and narrow nasal corridor. On the contrary, the trans-sylvian and subtemporal approaches require more superficial tissue manipulation, meaning that the long shaft impedes visualization and ergonomics (due to awkward hand positioning). Future iterations, with shafts of variable lengths and shapes, may improve performance in these approaches (Figure 5).

Beyond shaft-related concerns highlighted by the trans-sylvian and subtemporal approaches, there were other reported limitations (Table 2). Pertaining to the end-effector, these included insufficient grasping forces and tooltips that were too large or sharp, with limited articulation in certain planes. In addition, surgeons reported that the portfolio of available end-effectors should be expanded. Limitations relating to the controller included nonintuitive trigger and joystick functionalities (reported by 1 surgeon) and the shape of the handle. Design changes already in the pipeline (Figure 5), which include shorter, angled shafts, a wider variety of end-effectors with enhanced grasping force, and refined control interfaces, may address these limitations.

Overall, the handheld robot displayed useful characteristics across a variety of endoscopic skull base approaches, suggesting that it has the potential to overcome some of the limitations of currently available endoscopic instruments and thus expand the boundaries of what surgeons can safely reach by endoscopic means.

### Comparison with the Literature

As recommended by the IDEAL colloquium's frameworks for the translation of surgical robots, devices must be deemed safe, feasible, and acceptable in the early stages of translation.^16^ Therefore, preclinical studies should consider several stakeholder perspectives including the device, clinician, patients, and the system. Regarding the device domain, the IDEAL colloquium recommends the publication of both technical and clinical data, as well as transparent documentation of changes to devices. The current work addresses this recommendation. Despite significant advances in surgical robotics, the translation of such systems into skull base surgery has been limited.^17^ Beyond robotic exoscope holders^18^ and teleoperated robots capable of drilling the lateral skull base,^19-23^ the DaVinci system (Intuitive Surgical, Inc) has been the main device investigated for its feasibility in accessing the skull base, mostly in preclinical and early clinical stages of translation (IDEAL stages 0/1).^24^ These investigations have interrogated the DaVinci's capability to access the skull base through anterior and anterolateral approaches, including transoral,^24-26^ transmaxillary,^27,28^ transnasal,^29^ supraorbital,^30^ and transorbital^31^ approaches.^32^ Yet, despite the earliest studies being published over a decade ago, the DaVinci has not been accepted into the operative surgical landscape. Principally, this relates to the port size of the DaVinci, which has thus far been too large for the narrow natural corridors commonly used to access skull base lesions. In addition, the operating footprint of the DaVinci and friction associated with instrument changes places significant strains on the operative workflow. The potential consequences of this are nontrivial, as common tasks (such as bony drilling) or rare events (such as dealing with inadvertent vascular injury) may increase operative times or risk patient harm, respectively. Alternatives to the DaVinci include teleoperated continuum robots, consisting of thin, flexible, tubular shafts with interchangeable end-effectors capable of tissue manipulation.^33^ Although preclinical studies have validated some of these designs,^34^ continuum robots present issues related to sterilizability, controllability, and the need for a support base, which may limit distal-end dexterity and force delivery.^33,35^ Indeed, as highlighted in the literature^17,26^ in order for surgical robots to fulfil their theoretically disruptive potential and achieve acceptance in endoscopic neurosurgical practice, they must be designed around the inherent constraints posed by the confined anatomies and dense operative workflow associated with endoscopic neurosurgery.

To the best of the author's knowledge, the current robotic prototype is the first to demonstrate feasibility across 360° skull base approaches. The principal advantage of this system includes its simple, handheld design, which enables not only a minimally invasive means by which to manipulate tissue through narrow corridors but also a seamless integration into the surgical workflow, as it is a device which can be picked up and used with the same ease as any other instrument. As demonstrated in this study, such a device is not only versatile (and thus applicable across skull base approaches) but also offers unique advantages related to workspace reach in confined spaces.

### Strength and Limitations

Strengths of this study include the transparent description of device limitations, which enables a chronological account of the device's translation, aligning with guideline recommendations.^16^

This study has several limitations. First, the number of neurosurgeons evaluating the device was relatively small, and the data collected were primarily qualitative and subjective in nature, which may limit the generalizability of the findings. However, given that the primary aims of the study were to assess feasibility and usefulness, the reliance on qualitative feedback was appropriate, as these metrics inherently depend on user perception rather than objective performance measures.^2^ Moreover, none of the surgeons in the cohort had prior experience with the robotic device, and the potential influence of the learning curve was not formally assessed. Nevertheless, the finding that the device was still deemed useful is consistent with previous studies suggesting that the learning curve for its use is relatively short.^3^ Finally, there is the potential for bias, as some of the coauthors have disclosed relationships with the device manufacturer. Efforts to mitigate this bias included the use of independent surgeons to conduct the cadaveric evaluations and contribute to the relevant sections of the article.

## CONCLUSION

In this study, we performed a cadaveric assessment of the feasibility and usefulness of a handheld robot across endoscopic skull base approaches. We demonstrated that the robot was capable of safe tissue manipulation across endoscopic endonasal and transcranial approaches, which is a first in neurosurgery. In addition, we highlight useful features emerging from the device's simple handheld design, which includes an increased workspace reach and useful tissue manipulation capabilities. Overall, this study supports the potential of handheld robots to overcome some of the instrument-related limitations in endoscopic skull base surgery yet also highlights the need for iterative development to optimize instrument design and functionality.
